# 

**Published:** 1896-03

**Authors:** 


### CONTENTS.

ORIGINAL COMMUNICATIONS—
PNEUMONIA. By J. C. Olmsted, M.D., Atlanta, Ga. 1
THE TREATMENT OF PNEUMONIA. By James B. Baird, M.D., Atlanta, Ga. 9
THE ENDEMIC FEVER OF THE EASTERN SHORE OF VIRGINIA. By Geo. W. LeCato, M.D., Wachapreague, Accomac Co., Va. 14
THE SURGICAL TREATMENT OF DYSENTERY. By E. L. Patterson, M.D., Barnwell, S. C. 23
SOCIETY REPORTS—
ATLANTA SOCIETY OF MEDICINE 27
CORRESPONDENCE—
NEW YORK LETTER 36
IN MEMORIAM—DR. ROBERT BATTEY 42
IN MEMORIAM—DR. WILLIAM S. ARMSTRONG 43
EDITORIAL—
VOLUME THIRTEEN 47
DR. WILLIAM S. ARMSTRONG 48
WHAT'S THIS? ANOTHER "CONSUMPTION CURE"? 50
ROENTGEN'S DISCOVERY 52
SELECTIONS AND ABSTRACTS—
GENERAL MEDICINE AND THERAPEUTICS 54
MEDICAL ITEMS 63
BOOK REVIEWS 67
ADVERTISERS' INDEX TO ADVERTISEMENTS xiv
MEDICAL ITEMS—CONTINUED x, xi, xii, xiii
CLASSIFIED INDEX TO ADVERTISEMENTS xxxii

